# Thinking action as a performative and participative mental awareness

**DOI:** 10.3389/fpsyg.2023.901678

**Published:** 2023-05-02

**Authors:** Renatus Ziegler, Ulrich Weger

**Affiliations:** Witten/Herdecke University, Witten, North Rhine-Westphalia, Germany

**Keywords:** thought experiment in philosophy, phenomenology of thinking, mental activity, mental performance, shifts of conscious experience

## Abstract

This paper seeks to evaluate experiential facets of thinking action using first-person phenomenological methods. We begin our considerations using a simple mathematical proof as a case study—and also employ phenomenological contrasts between different types of thinking. They reveal that thinking actions produce performative insights rather than dispositional or remembered knowledge. This distinction allows us to introduce a new mode of thinking that is different from most known types of thinking, namely pure thinking action. The performative nature of this pure thinking action is participative and receptive with respect to concepts and has the quality of being persistent and coherent during its episode of action. Moreover, it is the often unattended source of thinking everyday life.

## 1. Introduction

Thinking is an inherent part of our daily life. In most cases it just happens to us, meanders along its own paths, and we become aware of it only when helpful flashes of insights or associations appear that carry our thoughts further and feed our reflections. However, in some cases, we need to take care of it in more systematic ways or think something through more deliberately. Yet, once again, what stands out for our consciousness in such a process are the results achieved, not so much the process itself.

Arguably, at the center of all types of conscious thinking there is *reflection*, i.e., thinking *about* given observations as well as thinking about thinking experiences accessible *after* performing thinking actions. Reflection draws on the two sources hinted at above: first on associations, memories, examples etc.; secondly, it draws on what has been done and experienced in thinking actions. Thinking action itself, as it will be discussed in this paper, is no reflection, but an explorative and experiential bringing about of conceptual relations by active thinking performance. However, in everyday thinking consciousness it appears in most cases *after* such actions, making us somehow aware that we *just recently did think* actively.

To be sure, thinking has been studied from many different perspectives. Thinking as an active process, as an action persisting for a certain period of time, however, is not evaluated on a regular basis (exceptions exist, see for example Burge, 1998; Proust, 2001, 2013; Buckareff, 2005; Soteriou, 2005, 2009b, 2013; Peacocke, 2007, 2009; Gibbons, 2009; Korsgaard, 2009).

A preliminary definition of thinking action runs like this: Pure thinking action is a performative action, a focused productive thinking within pure conceptual relations. Purity in this sense means being independent from factors outside active thinking performance (such as the involuntary or automatic popping up of associations and the like) as well as the conceptual content being independent from words, language in general, mental images etc. As to the content of thinking actions, namely concepts and conceptual relations, more on this subject has been written elsewhere (Ziegler and Weger, 2018, 2019).

Our current study is primarily concerned with thinking actions that are focused on types and conceptual relations (as in the definition of a triangle in the Euclidean plane) rather than with tokens (comprising statements such as: it rains in London, Grosvenor Square; there is cheese in my fridge). Available studies on this kind of thinking are rare (for an exception, see for example, Anderson, 2016, 2018) which is why we see a particular need in this direction. In addition, we work with the hypothesis that pure thinking action is not based on the use of words, sentences, or the like (these may be just there, parallel to it, but without determining its conceptual content)—making it elusive and difficult to observe to begin with. Some authors have already pointed to modes of thinking that are not guided by words (Jorba and Vicente, 2014; Lohmar, 2016). Some among them emphasize the role of concepts in determining the role of words etc. as we do (Pitt, 2011; p. 151; Nes, 2012; p. 103). But by and large, our understanding of pure thinking action as we understand it here remains underrepresented in psychological research. This is unfortunate because this type of thinking is something of a blueprint or birth-place of the other (type II) thinking.

The main issue then that this paper takes up is to show *first* that thinking action exists and, in particular, may be accessed and evaluated by phenomenological methods. *Second*, it turns out to be crucial that we are aware of the fact that thinking action may be contrasted distinctly from other known types of thinking. The latter means that we need to delve deeply into other, more common types of thinking in order to make explicit, by *contrast*, the characteristic features of thinking performances as mental *actions*.

While just thinking we often forget that *we* are doing it and that *we* are performing reflections and the like. Hence, in accordance with the first thesis this paper takes up, namely the possibility of evaluating thinking actions using phenomenological methods, it is important that it does not suffice to just *do*, for example, thought experiments or mathematics, but to *notice* and be *aware* of what kind of structural transitions occur while pursuing these pure thinking actions—in order to note (and avoid) potentially confounding intrusions from other kinds of thinking. Some of these issues are discussed in cognitive phenomenology (Bayne and Montague, 2011a; Breyer and Gutland, 2016b) and within the field of mental action and mental agency (O'Brien and Soteriou, 2009). However, the phenomenology of thinking *action* is rarely taken into account; sometimes it is only referred to in passing (Bayne and Montague, 2011b, p. 14–15), sometimes it is not mentioned at all (Breyer and Gutland, 2016a), sometimes it is explicitly excluded (Chudnoff, 2015, p. 80).

In order to meet this challenge, it is proposed here to tackle the experiential facts of thinking action by using first-person phenomenological methods (for a discussion of the reliability of introspection see Bitbol and Petitmengin, 2013a,b; Gutland, 2018b; Hackert and Weger, 2018; Weger et al., 2018a). Hence, Section 2 presents an example that encompasses—in a first step—important facets and features that need to be experienced individually, shared and integrated into research on the phenomenology of thinking action. This provides us with the experiential basis for many of our later excursions and considerations.

We now give a short overview of the main steps of this paper. With Section 3 on the phenomenological analysis of the said example, we emphasize that this paper is a contribution toward the description of the phenomenology of mental agency concerning thinking actions, and not about theories of mental actions or thinking in general. Hence, relevant experiences in this rather uncommon or under-appreciated field of research are described in relevant details: They make explicit what we mean by accessing thinking action. However, the aim is not just to describe these experiences, but to provide particular type experiences, namely detailed descriptions of experiences that can be shared intersubjectively at the type level and that are comparable with other research in this field. This is something to be learned from Husserlian phenomenology where the objective is not to collect endless descriptions of token experiences, but to identify invariant, essential structures (Gutland, 2018b). The main results of Section 3 are: Thinking action is a goal-oriented thinking performance guided by conceptual entities; it has two main functions: first, the productive capacity to arrange concepts according to their own rules and second, a receptive participative awareness of conceptual relations.

In Section 4 we review some core objections which might be at the forefront of the issues that readers concerned with the phenomenology of thinking action expect to be discussed. Section 5 outlines some characteristic elements of this phenomenological analysis which guided us in our introspective approach. Particularly it exposes what it entails to access thinking action, namely to take into account peripheral layers of thinking, in particular pre-reflective experiences, by extensions of our awareness.

In order to get a deeper and more nuanced view into thinking action, we now contrast this process extensively with other types of thinking, namely knowledge (Section 6), routine thinking (Section 7) and associations and flashes of insight (Section 8). Based on these contrasts, Section 9 presents an explication of our new thinking mode, namely pure thinking action introduced above, and juxtaposes it with Type1/Type2 modes of thinking.

Section 10 tries to answer the question: What exactly is pure thinking action? It draws together our main results by giving first a short summary of important types of thinking discussed in this paper; second, it presents an integrated overview of the most relevant features of our new mode of thinking, taking into account the results of the phenomenological analysis from Section 3 as well as the features gained from the phenomenological contrasts detailed in Sections 6–9. These main features are: Pure thinking action is embedded within all other types or modes of thinking discussed in this paper; it feeds these other types of thinking with conceptual content *after* being performed (which we are normally unaware of); it is explorative by its nature; it is initiated by a goal-setting thinker; it encompasses awareness in a participative and receptive mode; and it is consistent and persistent in its performative contribution.

Building upon the above considerations, we shortly discuss our approach in the light of some other approaches to thinking in Section 11. Section 12 draws relevant conclusions.

## 2. Experiential approach: an example of thinking action

Considering the main of this paper from Section 1 and phrasing them as a question we ask: can thinking action be accessed and, if so, what are its main features in contrast to other types of thinking? The following mathematical example goes a long way toward answering this question. Some preliminary results are presented in Section 3, taken up, advanced and expanded in Sections 6 to 10.

Why a mathematical example? Our focus is not on mathematics in particular, but on thinking action in general. We contend that, within mathematics, pure thinking actions with respect to pure concepts are simpler and easier to perform (and hence to access and assess) in an exact manner than in any other field, as for example in philosophy (logic, metaphysics). The questions of what is, and what is implied by, the purity of concepts have been explored in detail in other papers (Ziegler and Weger, 2018, 2019).

The example is about the proof that the sum of all angles of a triangle in the Euclidean plane is equal to 180°. This example serves several purposes: Firstly, to consider and then experience the presence of pure thinking action within this geometrical proof (namely to experience a mode of thinking that has been mostly overlooked, as explained in Section 9). Secondly, to realize what this thinking action, namely thinking in pure concepts, consists of, in particular in contrast to just gazing at or acknowledging the presence of specific geometrical figures or delving routinely into proofing the theorem. Thirdly, this example is the basis of the following phenomenological analysis in Section 3 (as well as of some considerations later on) which demonstrates some specific qualities of pure thinking actions. To serve as this basis, the example has to be actively performed by the reader, not just read through or simply acknowledged as such. There needs to be an experience of thinking action in the here and now in contrast to having some thoughts or memories of past experiences *about* thinking actions.

Some effort is needed to carry out the proof in our example, which encompasses several different steps. The example as such is not important, there are other possibilities or variations of it. Our aim is to present a specific cognitive task in which just one flash of insight is not sufficient; a process of interconnected insights is required to achieve an autonomous overall understanding. In this geometrical example, the main interest lies *not* in the various mental pictures or images, representing tokens rather than types, but in the conceptual relations they represent or that hold *between* them. Individual mental images may *point* or *refer* to universal conceptual relations but they do not directly convey conceptual qualities in the first place.

**Figure 1:**
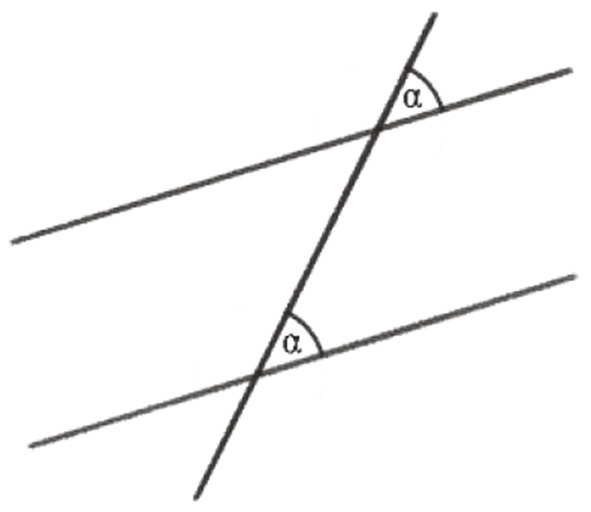
Corresponding angles α.

**Figure 2:**
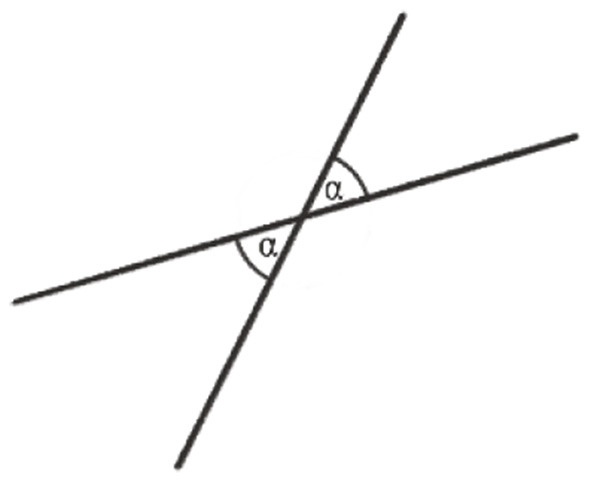
Opposite angles α.

**Figure 3:**
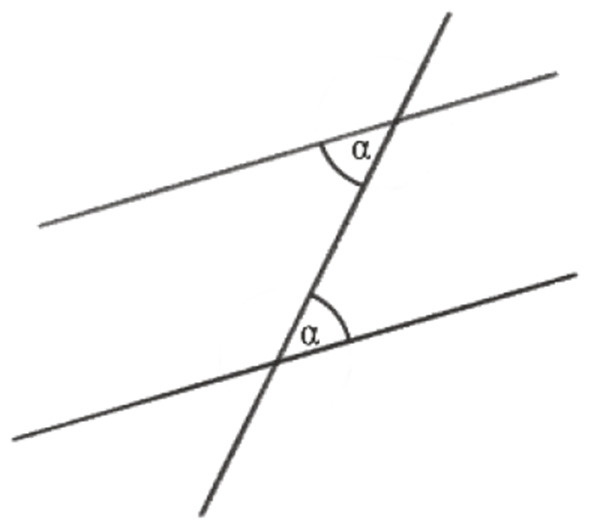
Alternate angles α.

**Figure 4:**
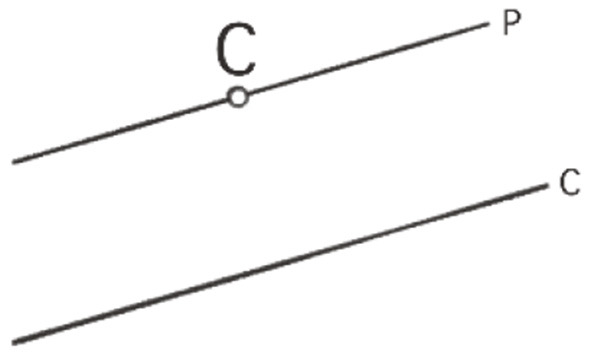
Parallel postulate: There exists exactly one line *p* through *C* not lying on *c*.

As said above, the following example is about the proof that the sum of the angles of any triangle in the Euclidean plane is equal to a straight angle or 180°, the full angle being 360°. This requires some preliminary insights or premises for geometrical relations in the plane: (1) There are parallel lines. (2) Any line intersecting two parallel lines has equal corresponding angles (Figure 1). (3) Together with the equality of opposite angles in one vertex (Figure 2), we have the equality of alternate angles (Figure 3). (4) Given a line *c* and a point *C* not lying on it, there is one and only one line *p* parallel to *c* through *C* (Figure 4).

**Figure 5:**
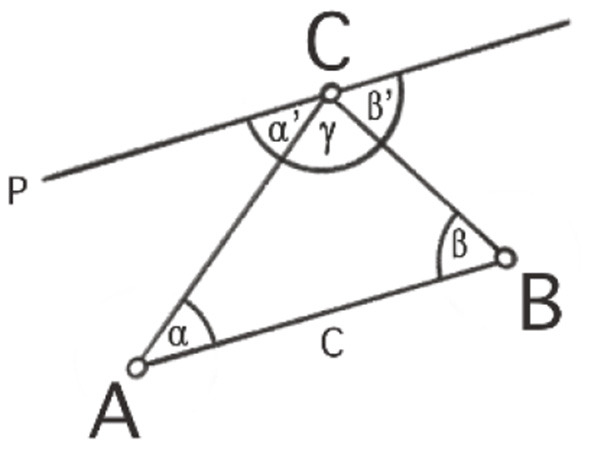
Triangle with vertices *A, B, C*, angles α, β, γ, alternate angles α, α' and β, β' and parallel line *p* to *c* through *C*.

Now take an arbitrary triangle with vertices *A, B, C*, and angles α, β, γ (Figure 5). Draw the line *p* parallel to *c* through *C*. The angles adjacent to γ on both sides, namely α' and β', are alternate angles of α and β respectively, hence α' = α and β' = β. They sum up in *C* together with γ to a straight angle:


$$\begin{array}{l} 180^{{}^{\circ}}=\alpha^{\prime }+\beta^{\prime }+\gamma =\alpha +\beta +\;\gamma . \end{array}$$

Since we did not use any particular idiosyncratic details of the triangle *ABC* in question (no specific angles or lengths), we can conclude: *The sum of all inner angles in an arbitrary triangle in the Euclidean plane is equal to a straight angle*.

## 3. Phenomenological analysis of our experiences with the example

The following descriptions concerning the example above in Section 2 are not descriptions of experiences of thinking in tokens or examples, but rather of experiences of thinking in types, or more succinctly, in concepts and conceptual relations within thinking actions. They therefore represent generalized, and in this sense artificial, reconstructions that serve primarily to illustrate what the authors of this paper want to share with the reader. These descriptions work toward the comparability and translational quality of our approach with other researchers working on these topics. The main purpose is to direct readers' attention to their own thinking experience since this is the only source available. Hence these descriptions are not intended as main evidence that guarantees the legitimacy of our contentions. Readers find such legitimacy only by using their own experiences as a tool to verify what is proposed in this paper. If readers cannot notice in the first instance what we have found and described here, they might try again and keep in mind that thinking action is not a capacity we can naturally draw and reflect on but something that has to be trained continuously and reactivated each time we want to experience it.

The following observations and reflections are an organized summary of experiences of performative thinking actions gained by both authors. They illustrate what we mean by accessing thinking action as proposed at the beginning of Section 2. We conducted these actions separately; the first author worked out the examples, went through these thinking experiences for a long time and did them more than 50 times (each session takes 5 to 10 min); the second author followed his instructions and further explored the field on his own. We then compiled the results and evaluated them conceptually by reflecting about them and writing them down.

The following part describes in more detail the method we applied. We first merely thought through the example several times and having completed this process, reflected about it afterwards (see Ziegler and Weger, 2018). After several cycles of this process, we were increasingly able to notice thinking experiences during the performance of thinking actions. This includes the extension of awareness focused on the qualities of conceptual content to begin with; and then on the performative experiences guided by the exploration of conceptual relations. Later on, these experiences (that is: the descriptions thereof) were collected and organized by both of us according to the noticed characteristics or qualities of experiencing pure concepts and thinking actions. With these characteristics in mind, thinking actions were performed again and assessed against the former results. That is, we compared the former descriptions with the new ones. Where differences remained beyond confirmations, we adjusted and enhanced our descriptions by gaining new specifications from performing again an experience of thinking action. This was done several times until we reached agreement on the main features of thinking action as outlined below. Our points of reference, or standards, for adjustments and correction were always the direct and noted experience we had from thinking actions, not just from any description of it.

The example from Section 2 can be analyzed on several levels: (1) First, one needs to specify the subject of the investigation, namely, analysis of the proof for the sum of the angles of an arbitrary triangle within the Euclidean plane. This is what one intends to think about. As soon as one is prepared to do this, several things start to happen: memories, mental images, words etc. might pop up, representing triangles, parallel lines, angles, propositions about angles and parallel lines, arguments, proofs etc. which are eventually gathered and collected for the purpose of thinking about them. We might speak here of occurrent (unordered) thoughts or mental images which carry with them beliefs that are based on past experiences.

(2) In this paper however, we want to focus on pure, productive conceptual thinking action. This means, we do not want to search for an insight that depends on what we already know or remember, but on what we can actually perform. This means that all our knowledge and memories are only the starting material for our active conceptual insight, namely for the shift from everyday type of thinking to the type of pure thinking proposed in this paper. If we want to give our thinking the shape of vigorous action we need to explore some surrounding concepts that might lead to the intended result, the main goal, namely the said proof (Buckareff, 2005). Some effort is required to think through the relevant concepts in order to execute a directed, controlled and voluntary thinking action (Proust, 2001, 2010; Soteriou, 2005; Peacocke, 2007). For example, are the concepts we considered necessary or sufficient as a set of concepts to prove the theorem (e.g., one needs the concept of a right angle only indirectly: a straight angle is equal to the sum of two right angles)? This means, the goal-oriented action needs to be persistent through the entire argument: it has to carry us through, although different concepts are involved; one needs to find transitions from one concept to the other—always keeping the main goal (the proof of the triangle theorem) in mind and staying committed to monitoring the process (Proust, 2010); otherwise one gets lost and does not find the correct argumentative path or is diverted to different, even non-geometrical subjects (Weger et al., 2018b). In other words, this goal-oriented thinking is participative with relation to the conceptual realm. This participation means that pure concepts are an experiential reality if and only if such a thinking action is performed, that is, only during such action.

To come back to the details of our example from Section 2: looking back after completion of the actual thinking process, one may observe that the decisive step in the whole argument is the step to introduce the line *p* in *C* parallel to the base line *c* of the triangle *ABC* (Figure 5). Having realized the necessity, the existence and the uniqueness of this line, all other pieces can be put together: the conceptual facts that corresponding and alternate angles depend on parallel lines and on the straight angle representing the sum of all three angles can now easily be accessed. From this point on, everything seems necessary, one knows how and why the concepts are connected, there is no arbitrariness. We are now in the position to autonomously arrange the entire argument by ourselves as presented above in Section 2. We can now weed out unnecessary side-lines (such as pondering on the intercept theorems), let go possible variations (for example, triangles on a sphere) and compose the argument so that everything can be woven together. Concentrating on the various conceptual relations involved in this argument, one might see the performance as merely revealing conceptual relations according to their own rules. This unveils a conceptual coherence that belongs to the subject matter rather than to the agency that performs the thinking action.

(3) However, if we extend or shift our awareness into agentive awareness [this term was first introduced by Bayne and Pacherie (2007), see also Proust (2009), and Mylopoulos (2017), for a defense] through active attention steering, a glance at the exact role of the performative action shows that the situation is more complex. In particular, if we look at the process of how we arrive at the final result, the first phases depend strongly on our own action: Particularly, one gathers and sorts out the elements that are needed for the proof of the theorem. This means, our agentive involvement is intense, we own the process as well as the content, we arrange the argument into a logical order such that it might even seem that we were constructing it (in contrast to discovering it). In the end, however, when all things are said and done, when we review the results culminating in the proof, our involvement seems to stop: In contrast with our earlier involvement, we now seem to be owned by the factualness of this small coherent cosmos of conceptual relations. Hence, we seem to have gone from active involvement to a merely receptive mode. This might then be the starting point for a period of post-evaluation.

To be more exact, however, this state of being owned by the factualness of conceptual relations, is only half the truth. In the first phase leading up to the proof, our sense of agency, our *performative persistency*, dominates our experience, but is already oriented toward the logical and geometrical relations relevant for this process: it is *participative* in terms of the conceptual realm. However, as soon as the whole proof stands before our inner eye, the sense of inner activity in arranging conceptual relations diminishes and gives way to a more *receptive state* that realizes the conceptual coherence in which we participate; that is, our inner action has transformed itself from arranging lines of arguments to seeing the whole conceptual arrangement.

Summing up the above, our thinking action appears to have two equally important structural aspects, or better, two non-separable functions: Firstly, a performative *capacity to arrange concepts* and arguments or whole processes from elementary conceptual facts, and secondly, a *participative awareness* while discovering the content of these elements and their overall conceptual structure. Thinking is then experienced both as (mainly) productive in its performative function and (mainly) receptive in its participative discovering function. However, there is no strict divide, temporal or otherwise, to separate the active and the receptive part; both functions involve the two aspects, depending on the viewpoint one takes on the whole action. One may shift in a controlled manner from one to the other and back. It is therefore appropriate to qualify this kind of pure conceptual thinking as an action which brings conceptual relations into experiential existence, and which discovers them by making them appear in our experience: it is constitutive for our having conceptual relations as an experiential reality.

(4) The importance of the receptive part of thinking, namely the self-sufficient consistency and invariance of the pure conceptual content, has been outlined elsewhere (Ziegler and Weger, 2019). In the following, the experiential-phenomenological qualities of the *performative part of thinking action* will be further studied in the form of two phenomenal contrasts (Chudnoff, 2015, Ch. 2; Bayne, 2020, p. 150–152).

For these contrasts one needs to differentiate between *performative insight* and *given knowledge* on the one side (first phenomenal contrast, Section 6) and between *performative action* and *routine thinking* on the other (second phenomenal contrast, Section 7). These are examples of structural differences between separate modes of thinking between which we may shift our awareness.

However, we first present a discussion of some objections against introspective accounts of thinking action (Section 4) and then describe some characteristics of our introspective account (Section 5). Both Sections provide some further important details of our method and may help the reader to work out specific pathways to first-personal experiences, in particular toward the experiences of thinking actions we are discussing here.

## 4. A review of some objections to introspective accounts of thinking action

The first objection against the possibility of introspective accounts of thinking actions that is discussed here is the “impossible split” objection. Thinking is an action we carry out ourselves; to observe it as a fact may entail an impossible split between the action one is carrying out and the simultaneous observation of this act. However, what is proposed is neither some kind of *observing* something as an object nor a reflection about the experiential content. What is required is to *be aware* of our thinking action *during* this action with an extended awareness that incorporates the fringes or margins of our consciousness. It can be described as an exploration that is *not* like using a torch or searchlight directed to what we want to experience, but experiencing something in a non-objectifying sense: It is a pre-reflective experience which forms the indispensable basis of any *later* reflections *about* it (otherwise, there would be nothing to reflect about—see also Section 5):

“Rather than switching the light on suddenly to see what the room looks like in the dark, it is rather exploring it in the dark, patiently, by feeling, with precision and delicacy, a little as a blind person would do. It is not a matter of ‘looking at' one's experience but of ‘tasting' it or ‘dwelling in' it. This exploration is encouraged by a particular attentional disposition, which is both open and receptive. Unlike focused attention, which is narrow, concentrated on a particular content, this attention is panoramic, peripheral, open on a vast area. This diffuse attention is however very fine, and sensitive to the most subtle changes.” (Petitmengin and Bitpol, 2009, p. 378)

We remark in passing that Husserl (1976, p. 162–165) argues—against our suggestion—that in his perspective of phenomenology such experiences are necessarily objectified. In his chapter on “Mathematical Intuition”, Tieszen (1989, p. 86–87) argues along similar lines, although in the different context of the construction or intuition of mathematical objects.

A second concern against the experiential grasp of thinking actions is that this experience might somehow interrupt or immobilize the process of the thinking action. But this is not the case, since we are not exploring something far away, foreign or opaque, but something manifestly present just within thinking action, something we are commonly not aware of in our everyday thinking life. What is required, is a shift in the quality of attending. Again Petitmengin and Bitpol (2009, p. 381) have argued against this concern quite succinctly:

“Far from disrupting it, freezing it or shrinking it, it seems that an increased consciousness of experience makes it more efficient, more fluid and meaningful, contrary to what indeed happens in the attitude that would consist in trying to consider oneself as an object. Entering into contact with our experience does not divide us into two but gives us back our entirety, our integrity.”

One may add that the *post hoc* knowledge about what we experienced during thinking action is reliable, that is, reflects what really happened, since there is no direct evidence to call this into question: We do not experience the transition from thinking action to *post hoc* acknowledgment of it as something corrupting, or substantially altering the content we experienced other than its active vs. passive presence. We are able to assess this and put it in an accessible form that takes up the generalizable features of our subjective agentive involvement, in particular, type experiences rather than several token experiences.

## 5. What are the main characteristics of introspective accounts of thinking action?

The primary aim of introspective accounts of thinking action, then, is to access and encompass these more peripheral layers of experiences that are located on the fringes of our consciousness and accompany focused (narrow) awareness on thinking action. It is certainly the case that these realms might be “concealed by our fascination for the objects of experience” and that they are “also masked by our preconceptions and beliefs” about such kinds of experience (Petitmengin and Bitpol, 2009, p. 384). However, as was pointed out above and is discussed further on, these experiences may be unearthed and integrated into our reflective awareness through a particular attentional practice (see also the discussion of this subject in Anderson, 2018, Ch. 4).

Another way to characterize the capacity of introspective accounts is the notion of *pre-reflectivity*. Reflective actions would not be possible without some kind of pre-reflective experience. Reflective assessment of what we have experienced in a non-reflective mode presupposes that there was an experience that was already inherently pre-reflective: “without this, the ability to re-appropriate past experience after the event would be inexplicable” (Gallagher and Zahavi, 2013, p. 56). This kind of experience implies immediacy in the sense that one is aware of such experiences without first reflecting about them, they are a pre-condition of knowledge: "Experiential episodes have [...] a first-person ontology from the start, i.e., even before the subject acquires the conceptual and linguistic skills to classify them as his own” (Gallagher and Zahavi, 2013, p. 43). This implies further that what “is needed if we want to ‘observe' the thinking process is not consciousness *of* what we do when we think, but consciousness *in* what we do when we think.” (Anderson, 2018, p. 61) One may add: We need a consciousness of *how* we experience thinking actions while we are performing these actions—prior to any kind of reflection *about* it.

Another important aspect was pointed out by Korsgaard (2009, p. 32) she observed that for the capacity of reflecting, there needs to be a “space of reflective distance” such that we are able to exert a kind of control over what and how we are reflecting: “we must step across that distance” such that we can be “active, self-directing”.

## 6. First phenomenal contrast: having knowledge vs. performative insight

Up to this point, we analyzed essential features of thinking action using only the example from Section 2. We now need to go further because thinking action is much richer than what has been extracted from our example so far. We emphasize that thinking action in general and in its details in particular is mostly overlooked because other types of thinking are in the forefront of our consciousness. It may therefore be necessary to look at more common types of thinking and use them, by way of contrast, to get a clearer idea of the specific features of thinking action.

We argue that to perform and be aware of an instance of thinking action is one thing but to be able to contrast it *in detail* with other kinds of thinking is another. Hence, we take up a methodological tool from cognitive phenomenology and discuss three phenomenal contrasts. The first, within this section, is concerned with having knowledge against performative insight; in essence, it shows that performative insights from thinking actions are the overlooked source of a substantial part of our thinking content (knowledge). The second contrast (Section 7) explores routine thinking relative to focused productive thinking action as described in Section 4. It shows that routine thinking may be overcome by a focused exploration of conceptual relations through thinking actions. Since associations and flashes of insight form an important part of what we usually consider thinking to be, they are dealt with in Section 8 and are put into perspective: they are contrasted with our approach to thinking action. It turns out that they do not belong to thinking action as we understand it here.

To begin with, knowledge can be understood as the result, the outcome that arrives as we finish our thinking process about the sum of the angles of a triangle in the Euclidean plane. In this sense, such results are the source of most of our common knowledge. One may write this knowledge down, express it in some computer programming language, communicate it, remember it, reproduce it, preserve it in whatever fashion one likes.

Performative insight is different: It depends on presence, on our involvement, it cannot be preserved by whatever means. It ends with our performative action.

This difference between performative insight and knowledge may be illustrated by the following phenomenal contrast: In the example from Section 2, knowledge is involved at two points, namely *before* we delve into the proof and *after* we have finished it. First, the proof can only be executed if we know what a proof is about, if we know what lines are, points, parallels, angles etc.; we may even have some prior knowledge about how the proof of the angle sum theorem should look like. Second, after performing the proof, after completing it and looking back at what we have achieved, perhaps planning to write about it or communicate it by other means, we enter into an episode of evaluative control, self-probing and post-evaluation where we have testable knowledge of all relevant details and the series of steps needed for the proof. Since this is the most accurate and up-to-date knowledge we have at hand presently, we take the last situation (completed proof immediately *after* our performative involvement) as one side of the first phenomenal contrast; the other side is the performative action while we actually go through the proof according to the example in Section 2. Table 1 gives details of the main features or structural dimensions of this knowledge vs. the performative insight we produce and are aware of *during* the pure thinking process present in the proof.

1. First, we look at the dynamic or temporal quality of the proof performance (Anderson, 2018, Ch. 7; Bayne and Montague, 2011b, p. 26; Ziegler and Weger, 2019, § 5.4): The performative insight is dynamic in the sense that it evolves, something is brought into experiential existence that was not an experiential fact beforehand (namely the conceptual relations between parallels, angles and the triangle); it takes time to advance an awareness of them. During the thinking process, we realize that we came from some point that is still present at the fringes of our thinking consciousness and that we are finding our way to the next steps by some prospective foresight or anticipation. – In contrast, knowledge is static, fixed; there is, beyond our performative action, no time involved in gaining or having it, nor is there any kind of transformation or evolvement of content: it is just there as it is.
2. Within a pure dynamic thinking action, the specific relational facts are furthermore not given in the form of propositions about concepts (and predicates) as in our usual knowledge, loaded with truth values according to our beliefs in the form of propositional attitudes; the relational facts have to be formed, or better: discovered or excavated in the first place from pre-predicative and pre-propositional experiential facts, guided by our performative insight or understanding: We experience *why* they are true or not, for what reasons, not just *that* they are. – In contrast, knowledge has the quality of being additive and combinatorial, the concepts, predicates and propositions are arranged according to formal rules, using truth values (coming from our beliefs); on the other side, within performative insight, the experienced composition evolves according to the unearthed (that is, gradually expanding awareness of) conceptual contents based on insight and understanding.
3. As to knowledge, we might have a sense of ownership: it is our knowledge, we possess it; however, as we are aware of its intentional structure, we look at it, we are detached from it. – In contrast, performative insight, as opposed to a sense of ownership, comes with a sense of participation: we brought it into experiential existence, we are not detached from it but are rather in some performative engagement with it; we are inherently in it rather than looking in from outside. In those experiences of thinking actions, we are *immediately* aware of the source (experiential facts: conceptual relations) and the messenger (ourselves) of our insight. Unlike our knowledge, nothing just presents itself to us and appears as true or not: in performative pure thinking actions we have to work for these insights. This kind of thinking experience is direct, immediate and thus reveals the non-intentional nature of thinking actions. Levine (2011) claims, for example, that “it's a mistake to view thinking with understanding as a matter of interpreting one's own thoughts.” (p. 109). Why is this so? We do not think *about* something but *within*; we are immersed in our thinking action experience, not looking at something from outside: *This* thinking experience *is* performative insight. This makes us further aware that knowledge comes with a sense of factualness, whereas performative insight comes with a sense of productive agency.
4. Finally, the experiential qualities of the self are very different for knowledge and for performative pure thinking. The awareness of the self in having knowledge is thin indeed: we know that this knowledge is our knowledge in the sense that we are in possession of it. – In contrast, performative insight during active focused pure thinking, that is, thinking action, is intrinsically linked to the awareness that we ourselves are the source of action, the agents of this process, we have agentive awareness (Bayne and Pacherie, 2007): We own it in the sense of bringing it into experiential existence (Horgan, 2007, p. 8, Horgan, 2011, p. 65; Mylopoulos and Shepherd, 2020, p. 174–183). As long as we focus on content, this agentive performance might be only aware at the fringes, or margins of our consciousness but is nevertheless crucial for our sense of engagement. The whole process is in our hands in the sense that we are the agentive source that turns universal conceptual relations into individual or subjective experiential facts: We experience the universal within the individual.

**Table 1:** Characteristics of the phenomenal contrast between having knowledge vs. performative insight.

	**Having knowledge**	**Performative insight within pure conceptual thinking**
(1)	Static, given, fixed	Dynamic, brought about, variable
	Instantaneous, non-transformative	Evolvement in time, expanding awareness
(2)	Given beliefs, truth and falsehood	Insight, understanding
	Propositional	Pre-propositional
	Predicative	Pre-predicative
	Combination according to formal rules based on beliefs of their truth	Composition according to conceptual contents based on insight/understanding
	Belief of truth as a propositional attitude	Experience the reasons why conceptual relations are true
	Object oriented awareness	Extended awareness to fringes of consciousness
(3)	Product/result of pure thinking action	Performative source of propositional facts
	Unknown origin	Source and messenger known
	Intentional	Non-intentional
	Sense of factualness	Sense of productive agency
	Detached	Performative involvement
	Sense of ownership	Sense of participation
(4)	Self in possession of knowledge	Self as source revealing conceptual relations
	Self having knowledge	Self with agentive awareness of conceptual relations

## 7. Second phenomenal contrast: routine thinking vs. focused productive thinking

The second phenomenal contrast involves a learning process where we produce our knowledge by our own means and are open about how we arrived at it. Initially, as we perform the proof that the sum of the angles of a triangle is equal to a straight angle for the first time ourselves, maybe with some outside help to induce or enable our thinking action, we have a fresh, pristine experience of the coherence of all relevant elements in one grand overview: our thinking process does not depend on anything outside its present and persistent action: no procedural memory or memory of the relational structure is involved (see below for more details on memory), no authority, no tradition; this might even evoke awe, wonder or joy in seeing all these concepts brought together and arranged in a harmonious whole. Soteriou (2013, p. 266–268), seems to discuss a similar example. He concludes:

“The suggestion here is that one brackets one's belief by reasoning in recognition of a self-imposed constraint; and importantly, the reasoning one thereby engages in is actual (and not pretend[ed] or imagined) reasoning […]. This involves mental activity that is self-conscious and self-determined, but which is also epistemic, truth directed, and subject to epistemic evaluation.”

Turning now to the contrast (see Table 2): (i) Assume that we have executed this proof many times, we have developed some routine, we have preserved it even in our procedural memory. Having routine means that after some minor stimulus (for example, someone mentioning the triangle and its angles) we are able to perform the proof that the angles of a plane Euclidean triangle sum up to a straight angle. Characteristic of such routine thinking is, first, its reliance on some memories (working memory) and/or mental representations (words, sentences, symbols, images, diagrams) that guide and organize our thoughts. We need *not* understand what we do and why we are doing this, it just happens, using the sources of our procedural as well as our representational memory; we may even remember some narrative that comes with this proof and makes it easier to reconstruct it.

**Table 2:** Characteristics of the phenomenal contrast between routine thinking vs. focused productive thinking.

	**Routine thinking**	**Performative insight within pure conceptual thinking**
(i)	Reliance on memories and mental representations (words, sentences, symbols, diagrams); they determine the routine thinking process; narrow focus on known facts and reliance on procedural memory	Permanent reassessment of mental representations and memories: transforms them from given knowledge to elements determined by the performative thinking action; widening the awareness and at the same time focusing on the present understanding of all elements involved
(ii)	Reproduction, repetitive: no variation	No reflection on previous thinking activities; fresh approach involves diverse variations
(iii)	Parallel mind wandering happens and need not to disrupt the routine	Mind wandering disrupts pure thinking: needs to be overcome
(iv)	Boredom, disinterestedness, ongoing comparison with past experiences	Happiness, awe, thinking lives only in the present with no comparison to past experiences
(v)	Sound knowledge of relevant consecutive steps	Knowledge may be present but is not directly relevant; soundness lies in the focused performative action, in the performative consistency and coherence

(ii) Having routine shows itself in the same pattern of thought processes every time we call it up: the reproduction of the proof turns out as a repetition, no variations are possible without falling out of the routine (and starting a new thinking action).

(iii) The mind may wander away while we are still doing this proof and communicate it to someone else: we may simultaneously, while executing the proof, observe the clothing of the person we are talking to or ponder about our lunch menu. This does not necessarily disrupt the routine thinking process.

(iv) No wonder or awe is present, no feeling that we do this for the first time; there may rather be some boredom, some disinterestedness which goes parallel to ongoing comparisons with memories of similar past experiences.

(v) Routine is, at its best, sound knowledge, but not understanding. We need not understand presently what we are doing routinely; we just have to know which series of steps we have to follow through.

Now comes the difficult part: After having acquired our routine (maybe by some hard training work), is it possible to carry out the same proof again as if for the first time? Can this structural shift be carried out in a controlled manner? And what are the phenomenological differences?

To set the stage: Yes, it is possible, and the differences as well as the consequences are profound. To think the proof anew without falling back into the acquired routine means to work against or break up the five characteristic features of routine thinking outlined above. In our own experience, this is best done by delving into the conceptual details of the proof, trying to understand it right now in the present and finding out why and how it is convincing. This involves the gradually expanding ability to harness our wandering mind (Weger et al., 2018b) and deal with diversions and associations (see below).

The experiential consequences of doing so are as follows. Regarding (i): If we focus on the *relational conceptual structure* of the said proof rather than on the elements that are related to it (points, lines, angles), then all memories and mental representations, words, pictures, diagrams, symbols etc. need to be reassessed for their meaning; they need to be transformed from elements determining the line of routine thoughts to elements that are determined and controlled by the actual performative and participative thought process. In other words, they have to be relegated to the background of thinking as mere accompanying features. As to the relational structure, our *present* understanding guides our thoughts, nothing else. To put it succinctly: We understand everything performatively from this structure but still know nothing (in contrast to our knowledge of the related elements themselves). In other words: our actual structural insight does not depend on given, previous knowledge of this proof—such might be the result, but not the pre-conditions of the proof performance.

Regarding (ii): As soon as we know that we did this proof some time ago, we are back to the routine and out of the actual productive thinking process. The active thinking process, the thinking action, does not allow reflections on what we did earlier or might do in the future: it lives in the conceptual relations present in the thinking process (otherwise we fall out of this thinking action). This means that we can carry out this proof with slight variations each time we do it—or even make some big variations by considering triangles on a sphere where the angles sum up to an angle greater than 180°.

Regarding (iii): Mind wandering and diversions are serious threats to thinking action in the sense presented here: they disrupt the continuity of the thought process, stray from the relevant conceptual relations and as such prevent understanding or insight. Hence, mind wandering and diversions, including associations, are incompatible with focused active productive thinking.

Regarding (iv): The fresh execution of our thinking process, a thinking action, makes us feel happy and content every time we do it. We are highly interested in what we are doing, and boredom has no chance since we are not reflecting on past experiences nor are we comparing them with our present doing. We have done something exciting by ourselves in the presence, have gained pristine insight by our own means—not directly or immediately depending on, or determined by, another person or authority or past experience: it happens just now.

Regarding (v): Knowledge in the sense of given representations of elements of the proof or even the whole proof procedure may be present in the background of our mind while we perform thinking actions. However, these representations do not determine our insight in the thinking action. Insight may be gained by using these representations as some starting material, but it leaves them eventually behind and comes to a fresh understanding. The soundness of our insight depends on the focused performative thinking action—not on given or memorized knowledge. The overall thinking action is due to its performative consistency (Petitmengin and Bitpol, 2009, p. 400) and its performative coherence (Bitbol and Petitmengin, 2013a, p. 270).

## 8. Third phenomenal contrast: associations and flashes of insight vs. thinking action

One might argue that disruption of routine thinking is not primarily due to active performative thinking, namely thinking action, as characterized above, which refocuses our attention on to what we are actually thinking. Instead, it may be due to associations or flashes of insight (Gutland, 2018a, Ch. VI.2.4, p. 425–429). Leaving aside the kind of diversions unrelated to our ongoing thoughts (for example, if we remember the grocery list for the afternoon), we are left with something that intrudes on us, interferes with us with its own force against our intrinsic action (which might be welcome for different reasons, but not for the ongoing thinking action).

As such associations—due to their content—may connect well with our performative stream of thoughts, they can delude us into thinking that they are in fact a direct continuation of our own action, even if they are not—and carry us away by their own (not our) intrinsic force (Weger et al., 2018b). A difficulty with this realization (that associations and flashes of insight are diversions and not part of the performative thinking process or thinking action) lies in the fact that flashes of sudden (deep) insight might appear as an enhancement, as an enlightenment, as a continuation or even a climax of our thinking action. We welcome them, are happy with them, we need them. We do not want to dismiss them because they may be exactly what we worked for, namely unexpected variations of our thought process, deep insights or at least some as yet unknown associations. However, even if they are stimulated or triggered by our active conceptual phase, even if we experience them more often than not after an intense period of active explorative thinking, they have to be classified as something different: they are, at best, indirect or secondary outcomes of our action but not part of it in its essence: they may pop up or not. There is no intrinsic necessity in active performative thinking, or thinking action, that produces, or brings about or asks for, flashes of insight; they happen on their own account, not as an essential ingredient, or a compulsory consequence of performative thinking actions.

Important arguments for the foreign character of flashes of insight with respect to active performative thinking are the following: If we fail to integrate them into what we already know, in particular into a coherent explorative survey of relational content, they are lost, they pass by and become worthless. On the other hand, if and only if we embed them into one of our active streams of performative thinking, they may become fruitful. That is, we must make them part of our active thinking process in order to arrive at something that we can evaluate ourselves and that in the end can further our research.

It should be clear by now that this paper does not want to rigorously exclude this kind of sudden insights from any general account of thinking (on the contrary). But one may argue that they do not belong to the type of thinking that is the main subject of this paper—namely active performative thinking or thinking action—as long as they are not actively integrated into it.

This being said, it nevertheless seems that some kind of “flashes of insight” are experienced that appear to be the pinnacle of some more or less complicated performative thinking actions. Often, they occur *after* such performances during a time of relaxation. They may mark the ultimate success of actively understanding something instead of just passively knowing it. We think that this is indeed the case. But one should carefully differentiate the gentle *light* of insight during actively understanding something via a thinking action from the more dominant *flashes* of insight that take place without our immediate action. The first unfolds more or less gradually as we proceed along our performative thinking process: it is an intrinsic part of our productive thinking *action* or performance, it encompasses more and more of the whole structure until we have the overview we longed for. The latter, the flashes of insight, come over us from outside the thinking performance as such, like flashes of lightning appear from the outside with respect to our body (and our eyes particularly), and are, in their quality of appearance, *not* part of our thinking *action* in the more immediate sense of the word. Instead, they simply appear as an element that is foreign to our active thinking performance (however, not to our thinking in general).

## 9. Thought, reason, and reflection—Introducing a new mode of thinking: pure thinking action

In this Section we argue that, according to the research laid out in the foregoing, particularly concerning the contrasts in Sections 6 to 8, the psychology of thinking may require to consider a new mode of thinking, namely *pure thinking action*, a new mode to complement the conventional Type 1 and Type 2 thinking processes.

To begin with, Jorba and Moran (2016, p. 98) pointed out that in psychology, particularly in cognitive psychology, there is “a well-established division between unconscious and conscious thoughts on the basis of two different cognitive systems or processes that underlie thinking.” Type 1 thinking includes forms of reasoning that are passive, reflexive, spontaneous, unreflective, fast, automatic, behavioral and non-conscious. Type 2 thinking involves processes that are actively adopted, actively executed, rule-based, analytic, language-related or reflective and use hypothetical thinking and mental simulations as well as working memory (Evans, 2008, 2010; Frankish, 2010; Evans and Stanovich, 2013). This so called dual-process theory approach can be found in separate areas of psychology and philosophy, such as learning, reasoning, social cognition, judgment, decision making, and in the philosophy of mind under various different designations. This distinction goes well back into the history of psychology; however, apart from minor adjustments, it has been quite stable over time until today.

In philosophy, one finds this differentiation labeled as belief vs. opinion, or belief vs. acceptance. Most psychologists and philosophers think that Type 2 processes are based on natural language; in addition they contend that natural language in general serves as the medium of conscious, explicit thought. Moreover,

“many researchers now accept that it is wrong to characterize System 2 [=Type 2] reasoning as uniformly abstract, rule-based and logical. Explicit reasoning, they argue, may involve a variety of other techniques, including the application of heuristics, explicit associative thinking, manipulation of mental imagery and selective direction of attention.” (Frankish, 2010, p. 921; see also Evans, 2009; Stanovich, 2009)

A further important aspect of Type 2 thinking is the capacity of “cognitive decoupling,” a central feature of Type 2 hypothetical reasoning:

“In order to reason hypothetically, we must be able to prevent our representations of the real world from becoming confused with representations of imaginary situations.” (Evans and Stanovich, 2013, p. 236)

Evans introduces a further distinction, a further category of processes, Type 3, that is supposed to be responsible for initiating Type 2 processes and possibly resolves conflicts between autonomous (automatic) and analytic processes, and which have ultimate control over behavior (Evans, 2009). Along similar lines, Stanovich (2009, p. 67–72) makes a distinction *within* Type 2 thinking between the reflective and the algorithmic mind and contends that the reflective mind is at the top level, consisting of higher-level goals and higher thinking dispositions such as open-mindedness and willingness to engage in effortful thought, which regulate and shape our conscious reasoning [see the discussion in Frankish (2010, p. 922–923)].

From the perspective of this paper, the distinction between Type 1 and Type 2 thinking processes is indeed important. What has been called knowledge in the first phenomenal contrast is close to Type 2 processes, as is the case with routine thinking, involving some complicated routines that need our thinking attention, in the second phenomenal contrast. Associations, flashes of insight and simple thinking routines, however, belong to Type 1 reasoning. Closer inspection reveals that things are more complicated. The most important aspects of Type 2 reasoning seem to be reflexivity—based on working memory and language. However, this rules out what has been called pure thinking action above; such pure thinking action is neither of Type 1 nor of Type 2 (nor of Type 3 within Type 2), since it is neither passive nor language-related, nor associative, nor based on imagery.

Thus, the faculty of hypothetical reasoning as well as the engagement in higher level goals and higher thinking dispositions (open-mindedness, willingness to engage in effortful thought) *within* Type 2 reasoning outlined above, comes close to this pure thinking action, but there are still considerable differences.

In conclusion we may say that reflection, particularly, as a Type 2 process, draws upon several sources: it works with what comes to mind automatically, without effort, spontaneously, and integrates the output from these sources into the reflexive reasoning if and where applicable (see the discussion of associations and flashes of insight above in Section 8). Reflective reasoning also works with explicit memory, inference rules, logic, language, etc. However, and this is one of the main points this paper wants to suggest, it also draws on the *results* of pure thinking action although these results stem from a quite different thinking type. It has been outlined above why this type of thinking action eludes normal attention and that it takes quite an effort to remedy this situation. This is true even when we contend that reflexive thinking draws on the results of pure thinking actions in the form of representational modes of concepts, namely conceptual relations reduced to propositions and their relations.

The upshot of this observation is that the distinction of Type 1 and Type 2 reasoning is not complete: There is another type—rather distinct from Type 3 mentioned above, namely *pure thinking action*, that needs to be considered for a more comprehensive theory of what thinking entails. These actions are neither spontaneous nor unreflective events (Type 1) nor higher order reasoning processes based on working memory and language (Type 2). The latter type of thinking works with explicit memory, inference rules, logic, language, etc. However, and that is one of the main points this paper suggested repeatedly, it also draws on the *results* of thinking actions without us realizing that these results stem from a quite different type of thinking.

This is the reason for evaluating this type of thinking, namely pure thinking action, more thoroughly. Pure thinking action encompasses a controlled structural shift and a change of levels of consciousness from Type 1/Type 2 thinking to dimensions of thinking that are not covered by this theory.

## 10. What exactly is pure thinking action?

First, we give an overview of the most important types of thinking discussed in this paper (Table 3). At the center of all types of conscious thinking there is *reflection*, i.e., thinking *about* given observations as well as thinking about thinking experiences accessible *after* performing thinking actions. Reflection draws on two sources: first on associations, memories, tokens of thinking, examples, images, observations, mental representations etc. It combines, then draws conclusions, makes predictions, has knowledge and uses thinking routines and working memory. However, secondly, it also draws on what has been done and experienced in pure thinking actions which is often overlooked. Thinking action itself, as already outlined above, is no reflection, but an explorative and experiential bringing about of conceptual relations by thinking performance.

**Table 3:** Main types of thinking.

	**Occurrent thinking, type 1: thoughts happening to us**	**→**	**Reflection, type 2: thinking about thoughts**	**←**	**Pure thinking action: new type: awareness within thinking action**
**Content**	Associative relations, flashes of insight, mental images, memories, occurrent thoughts		Given knowledge (additive, combinatorial, propositions, predicates, conceptual relations attached to words, images, propositional attitudes)		Pure conceptual content, thinking in types (focused productive conceptual thinking)
**Activity**	No individual action, routines		Mixed activity: actions and occurrences		Pure individual action, active process, performative exploration, active performative insight (evolving during time), participative, discovering, sense of agency, self as source of action

Reflection about the results of former thinking actions as a starting point is a way to access this action. And then, after some practice, we take these results into a non-reflective focus—goal-oriented as a result of previous reflection—and thus envisage our thinking action in its entirety; in this process we extend our awareness from conscious conceptual content to the performing action itself.

Some results will now be drawn together, namely from the phenomenological analysis of the example (Section 2) in Section 3 and the results from the two phenomenal contrasts in Sections 6 and 7. They are integrated with our further considerations of flashes of insight in Section 8. These lead toward a comprehensive overview of the main features of pure thinking action and allow us to set this new mode of thinking apart from Type1/Type 2 thinking discussed in Section 9. These results achieve the character of statements that can be discussed and put into perspective with results from other research. What has been explored in this paper, namely focused productive conceptual thinking actions with their performative features, will now be abbreviated consistently with the term *pure thinking action*. As mentioned above, more has been said elsewhere about the content of thinking actions, namely concepts and conceptual relations (Ziegler and Weger, 2018, 2019).

(I) *Embedding*: Pure thinking action is preluded by, surrounded by and *embedded in occurrent thinking*, that is, in pre-performative and post-performative events; it is often—but not always—triggered, occasioned, or induced by knowledge, memories, associations, mental images, and mental representations. However, these factors neither determine its content nor its performative appearance. This means that they may continue as diverting factors or accompanying events on the fringe of our overall thinking consciousness but do not determine the content or performance of pure thinking actions. This content of pure thinking actions has its own experiential reality that does not depend on anything else (Ziegler and Weger, 2018, 2019); it is part of the experiential reality present in this kind of thinking [see also below (4) and (5)]. Thinking action is the frequently overlooked source of substantial parts of our common knowledge.

(II) *Explorative nature:* Pure thinking action as a mental action does not bring pre-specified conceptual content into experiential existence, but tries to pursue and explore, by its overall goal of conceptual awareness, the specifics of conceptual relations which do not emerge on their own. However, as such they constitute intrinsic conceptual constellation before, after or outside pure thinking actions or processes which are not constructed but revealed or discovered by our pure thinking action [see below (4)]. Pure thinking actions or processes have the character of explorative experiments where a process of experimental awareness is initiated with clear-cut initial conditions that are varied during the performative engagement. In this sense, pure thinking action is an awareness which is pre-predicative, pre-propositional, pre-inferential and pre-reflective as well as pre-intentional. We are aware that the latter qualities are traditionally applied to very basic, passively experienced, even unconscious facts. However, our contention is that these qualities may be applied to pure thinking actions as well. Even in such a simple example as the one in Section 2, these qualities are present: If we do not know the proof in advance (or ignore knowledge of it), we have to explore the relational features of points, parallel lines, angles, and planes in order to find the relevant elements for the proof. This exploration might bring us temporarily to some other structures, such as triangles within circles (for example, the Thales case with a right angle) or triangles on a sphere, before we come back to what we set out for.

(III) *Initiation and goal setting:* Pure thinking actions are initiated and governed or directed by overall goals, as for example the aim to incorporate into phenomenal awareness the proof of the angle theorem for plane Euclidean triangles. This may be transformed into a more general mode of exploration, where different and/or extended subject matters may be pursued and conceptually connected with each other without interruption. One example is the exploration of the conceptual relations with respect to segments on a line and their interrelations with the concept of triangle, circle, etc. (Ziegler and Weger, 2018).

(IV) *Conceptual awareness:* The performative conceptual awareness during pure thinking action reveals conceptual relations that are invariants of the actions of pure thinking performances. The individual act of pure thinking actions meets universal conceptual content and thus experiences *universals within the individual*, namely universal concepts with experiential qualities that are beyond time and space, since these relations have neither time-dependent nor space-dependent features. For example, the universal conceptual content of the proof of the triangle theorem in the Euclidean plane (where there are parallel lines and there is a unique parallel with respect to a point outside it) can be spelled out as follows: Given that corresponding and alternate angles in a line intersecting two parallel lines are equal, and given a line through any vertex parallel to the opposite side of an arbitrary triangle, then it follows that the angles of any triangle must always add up to 180°. Given this, it is obvious that the overall structure of the proof of the triangle theorem has no spatial or temporal features; it is sufficient and essential in itself, has its own inner connections which we experience when actively thinking it through. One might speak here of *participative insight* into the intrinsic necessity, or the essence, of concepts and conceptual relations. For the agent performing this encounter this comes with a sensation of clarity or light, which can be described as seeing something as transparent with the mind's eye.

(V) *Participative nature:* Pure thinking action is performative as it creates (that is, brings into existence) an experiential awareness of conceptual relations; it is participative in its ownership, in its engagement through insight and understanding. Being aware of the said proof by pure thinking actions means being part of it, participating in its structure, revealing this structure, discovering it, making it experientially available (in contrast to constructing it, inventing it, making it up). Pure thinking action has its own agentive phenomenology: we experience it as an individual force with its performative and participative awareness, with its active encounter of conceptual relations *within* its extended awareness from conceptual content to performative action.

(VI) *Receptive nature:* However, by closer inspection, participation as outlined in (5) above reveals another aspect of our pure thinking action or performance which we now can spell out more explicitly: Participation means that there is something that we participate in, which is not by our own making. However, and that is the important point that we now want to present, this participation happens only *while* we are performing and are aware of pure thinking action. It is a participation *within* active involvement, when we encounter something (namely pure concepts) which is revealed by this performance in its experiential existence but does not result from it. We wrote earlier in Section 3 in our phenomenological analysis that this might be termed a *receptive* mode of thinking action. However, this has nothing to do with the more common types of thinking such as knowledge, routine thinking etc. More clearly, it is a receptive mode *within* the performative action of pure thinking action. The action itself (besides being an action) also has the function of taking into account, receiving, acknowledging, “seeing” etc. conceptual content (we are not suggesting that these contents force themselves upon us by their own account). In other words: The receptive mode or aspect of the performative action of pure thinking is *relatively* receptive with respect to this action, not receptive in itself. The receptivity is not a sufficient hallmark of pure thinking action, but only a necessary ingredient. Hence, it cannot be separated substantially from pure thinking action but only distinguished conceptually from its pure performative aspect. If we want to find out if the conceptual contents “received” by this receptive mode are part of the performative action, we only need to check whether the conceptual content is revealed as something manifestly and intrinsically clear without reference to any previous or non-performative knowledge—if not, a different kind of reception has taken place (association, flash of insight, remembrance etc.).

(VII) *Performative consistency and persistence:* Pure thinking action has its own dynamic quality and persistence. Pure thinking action is not tied to only one concept or conceptual relation but works with transitions between them. This active persistence enables pure thinking actions to perform transitions from one concept to another without leaving the performative realm; this is in essence the sense of agency that includes the persistence of performance as well as the persistence of participation. The performative awareness which guides itself by staying engaged with its content leads to the performative consistency as well as to the performative coherence that qualifies pure thinking actions.

(VIII) *Performative contribution:* Pure thinking action ends by contributing elements to our knowledge: its transformation, or rather its fall, from active involvement to the static character of its results (which can be written down and communicated), adds to the environment that encompasses all pure thinking processes or actions: What we have thought actively beforehand belongs now to the starting material we may use and need to initiate a subsequent thinking performance. Thus, we are back to (1) and may start a new pure thinking process.

## 11. Thinking action in light of other fields of research

Mental action and mental performance in their dynamic quality within pure thinking actions have already been evaluated by phenomenological methods using first-person experiences (Anderson, 2016, 2018; Jansen, 2016; Ziegler and Weger, 2018, 2019). These evaluations take into account that such thinking processes are temporarily extended, i.e., they unfold in time (Bayne and Montague, 2011b, p. 26; Chudnoff, 2015; Jorba, 2015; Ziegler and Weger, 2019, § 5.4). This might shed some light on whether the dynamic quality of agentive experience within physical actions could be extended to cover mental acts.

Since thinking action is a multifaceted experience, first-person awareness has to be extended to the margins of consciousness: this has already been suggested by other authors (Mangan, 2001; Bayne, 2008, p. 108; Petitmengin and Bitpol, 2009; Mylopoulos and Shepherd, 2020, p. 169). Furthermore, several kinds of self-consciousness have to be taken into account, particularly of the pre-reflexive or pre-predicative kind (Gallagher and Zahavi, 2013, Ch. 3; Ziegler and Weger, 2019, § 6.2). This includes the capacity to generally enhance conscious awareness in thinking (Montague, 2016). This pre-reflective self-consciousness facilitates reflective self-consciousness but is not in itself a reflective self-consciousness.

Mental action and mental agency are major fields in their own right: This paper is not the appropriate context to address the diverse discussions and debates in detail, see for example (Soteriou, 2009a; Fiebich and Michael, 2015; Metzinger, 2017). For a short discussion, the challenge by Strawson, 2003; is taken up and confronted with our approach. Similar viewpoints have been worked out [see for example Tye and Wright (2011) and Vicente and Martínez-Manrique (2016); see also the discussion in Anderson (2018, p. 80–92) and Fiebich and Michael (2015, p. 685–687)]. One should not take the easy way out in stating that Strawson's point would be similar to what has been called “having knowledge” and “routine thinking”, or even associations and flashes of insight: Strawson leaves this option open by making sure that he considers only what he believes most thinking people do: “most of our thoughts—or thought-contents—just *happen”* (p. 228), namely, that people who believe “that much or most of their thinking is a matter of action are, I believe, deluded” (p. 231). However, one should take seriously his contention that “the role of genuine action in thought is at best indirect. It is entirely *prefatory*, it is essentially—merely—*catalytic”* (p. 231). Strawson is sure that there are actions in our thinking, but these are “acts of *priming*, which may be regularly repeated once things are under way, [they] are likely to be fully fledged actions” (p. 231), even attention is assumed to be “a matter of action” (p. 232).

Perhaps Strawson found it rather odd that we should experience something intrinsically existential, namely conceptual content, while performing a thinking action where the latter might rather suggest that we construct our thinking contents ourselves. However, paradoxically, this is exactly the case: The pure thinking action is the *conditio sine qua non* of experiencing pure concepts and conceptual relations *not* produced by the action in any respect (except their experiential appearance). This is another way of saying that pure thinking includes agentive and receptive aspects: bringing something into experiential existence *and* being aware of it at the same time.

To consider this further, we need to take into account what has been described in the Sections above: After some effort to capture what happens on the fringes of our consciousness (Mangan, 2001; Petitmengin and Bitpol, 2009), we may become conscious of our own agentive contribution toward the thinking action in the sense of the agentive awareness according to Bayne and Pacherie (2007), which does not impinge on the non-subjective essence of conceptual thought content (as in the mathematical example in Section 2). One has to consider something like “seeing with the mind's activity”, or “grasping” (Brown, 2004; Pitt, 2004, p. 10–11; Chudnoff, 2015, p. 39–40). This means that in thinking we are aware of universal conceptual relations that transcend our individual consciousness but nevertheless appear within it (Hopp, 2014). This is “not a matter of positing purely abstract ideality as metaphysically existent, but rather grasping or ‘seeing' the universal in the individual” (Froese and Gallagher, 2010, p. 89). For more on the content-oriented view of thinking, see Parsons (1979), Bealer (1993, 1998), Tieszen (2010), Chudnoff (2014); and Ziegler and Weger (2019).

Others took up the challenge of Strawson's claims by pointing to qualities of the thinking action that come close to what we present in this paper. Among those views on thinking action, the most appropriate and promising approach to evaluating thinking processes appears to be the goal-oriented view on mental action (Mele, 1997, 2002; Buckareff, 2005). This view takes into account that specific thinking tasks are performed while one keeps up with thinking activity in general. A thinking process is a complex undertaking with different phases and encompasses various tasks (Anderson, 2016, 2018); therefore a teleological theory and an appeal for trying is appropriate (Proust, 2001, 2010; Soteriou, 2005).

The sense of agency or sense of ownership cannot be discussed in any detail here, since this is a complicated matter (Gallagher, 2012, 2013; Mylopoulos and Shepherd, 2020) that needs to be pursued in further research on thinking action. However, let it be stated that it is the sense of agency that makes us aware that we are thinking and not doing something else (Bayne and Pacherie, 2007; Gallagher, 2012; Proust, 2013; Mylopoulos, 2017); in addition, one may have to take into account what is called agentive phenomenology (Pacherie, 2008; Jansen, 2016).

Another aspect that cannot be addressed further in this paper is the role of the self in thinking actions. It should be noted, however, that the role of human agency in thinking action, the issue of autonomy and self within thinking action has been barely researched (Guillot, 2016; Jansen, 2016; Jorba and Moran, 2016).

## 12. Conclusion

Thinking may in many cases just happen, filling our mind with memories, ideas, propositions and the like. However, there is another mode of thinking which in this paper is called focused productive thinking action, or short, pure thinking action which is the source of many insights we just have and do not know where they came from. This paper is also concerned with making us aware of pure conceptual relations—having their own universal status—which are not the product of our subjective self or the environment but nevertheless play an important role in the advent of our representational knowledge. Phenomenal contrasts serve to show that thinking actions produce performative insights rather than merely dispositional or remembered knowledge. Our presentation seeks to extend routine thinking into focused productive thinking actions that open up new perspectives. This extension presents shifts of awareness to new structural dimensions of the conscious experience of thinking. The performative nature of thinking actions is explorative, guided by overall goals, participative and receptive for concepts or ideas and has the quality of being performatively persistent and coherent during its episodes of action.

All this is intrinsically linked to our self as being the agent of this action and executing it by keeping up our thinking action with persistence and coherence. In the end this can reveal something about our self: We are capable of executing something that, as an action, does not depend on anything else other than our self.

## Data availability statement

The original contributions presented in the study are included in the article material, further inquiries can be directed to the corresponding author.

## Author contributions

RZ conceptualized this research topic and wrote the first draft using the methods of phenomenology of thinking. UW worked through the manuscript and contributed his expertise in the psychology of thinking. All authors contributed to the article and approved the submitted version.
